# The prognostic value of MKL1 in predicting breast cancer immune infiltrates and chemosensitivity

**DOI:** 10.17305/bjbms.2021.6306

**Published:** 2021-11-10

**Authors:** Yijia Hua, Mengzhu Yang

**Affiliations:** 1 Department of Oncology, The First Affiliated Hospital of Nanjing Medical University, Nanjing, China; 2 Department of Geriatric Oncology, The First Affiliated Hospital of Nanjing Medical University, Nanjing, China

**Keywords:** MKL1, breast cancer, immune cell infiltration, chemosensitivity

## Abstract

Megakaryocytic leukemia 1 (MKL1) acts as a transcription factor in the regulation of the immune system and is associated with cancer biology. However, its function in the infiltrating immune cells in breast cancer has not been explored. Our study aimed to analyze the expression of MKL1 in The Cancer Genome Atlas breast cancer dataset. The aim of this study was to evaluate the correlations between MKL1 expression, infiltrating immune cells, and immune control genes. Enriched signaling pathways and drug sensitivity analyses were also performed. Our results indicate that high MKL1 expression could predict better survival in breast cancer patients. MKL1 expression was associated with the expression and function of different immune cells, including T cells, B cells, natural killer cells, macrophages, neutrophils, and dendritic cells. The chromatin-modifying enzymes, cellular senescence, epigenetic regulation of gene expression, estrogen-dependent gene expression, and chromosome maintenance were differentially enriched in MKL1 low expression phenotype. Patients in the high MKL1 expression group showed sensitivity to paclitaxel, while those in the low expression group showed potential sensitivity for cisplatin and docetaxel. In conclusion, MKL1 might act as a potential biomarker of prognostic value for immune infiltration and drug sensitivity in breast cancer.

## INTRODUCTION

Breast cancer, known as one of the most common malignancies among women worldwide, raises concerns nowadays [1]. Aggressive proliferation and invasion, resistance to chemotherapy, and evasion of immune surveillance make breast cancer prone to relapse and metastasis [2]. However, with an improved understanding of tumor behavior, its microenvironment, and host factors, immunotherapy strategies have been promising for breast cancer treatment [3-9]. Therapeutic anti-tumor immunity is activated and the function of T cells is enhanced through targeting immune checkpoints [10]. Blocking naive CD4+ T cells from entering into breast cancer tumors also significantly decrease tumor-infiltrating Tregs and prevent tumor progression [11]. Moreover, the CD47-immune signature was also found to be related to the presence of tumor-infiltrating immune cells, such as T cells, dendritic cells (DCs), and neutrophils, and could predict the survival of breast cancer patients [12].

Megakaryocytic leukemia 1 (MKL1) is a transcription coactivator associated with the serum response factor. It controls the expression of cytoskeleton-related genes during development, morphogenesis, and cell migration [13], and plays a critical role in epigenetic regulation [14,15]. It was also indicated that MKL1 may exert specific effects on immune cells. For example, MKL1 deficiency impairs the function of the immune system and results in primary immunodeficiency. The migration and function of neutrophils are affected by the dysfunction of actin polymerization [16,17]. Recently, the relationship between MKL1 and human cancers has attracted wide interest. Various studies have shown that MKL1 may be associated with cancer cell development, progression, metastasis, drug resistance, apoptosis, and autophagy [18-22]. However, the relationship between MKL1 expression and breast cancer infiltrating immune cells has not yet been reported. Considering the critical role of MKL1 in both immune regulation and tumor development, the clinical prognostic value of MKL1 in breast cancer warrants further investigation.

Therefore, our study aimed to explore the relationship between MKL1 expression and the infiltration levels of immune cells in breast cancer. We also assessed the associations between MKL1 expression and a series of tumor-related immune cells, and the results were validated in an independent cohort, GSE158309 [23]. Our results showed that MKL1 expression was associated with the tumor microenvironment (TME) in breast cancer and could predict breast cancer patient chemosensitivity and survival status. This study revealed that MKL1 may act as a promising biomarker of clinical value.

## MATERIALS AND METHODS

### Data collection and processing

To study the expression of MKL1 in breast cancer and pan-cancer levels, the fragments per kilobase of exon model per million mapped fragments normalized expression datasets of mRNA sequencing of 1109 breast cancer tissues, 113 normal tissues, and 9951 other different cancers were downloaded from the UCSC Xena hub (version 2017-10-13, https://xenabrowser.net/) [24]. The genes in the expression matrix were annotated using the GENCODE v19 genome assembly. Matched updated clinical information for these samples, such as gender, age, disease-specific survival (DSS) status, and progression-free interval (PFI) time, were retrieved from The Cancer Genome Atlas (TCGA) Pan-Cancer Clinical Data Resource [25]. Patients with missing records were excluded from further analysis.

### Analysis of the infiltration levels of immune cells

To evaluate the association between tumor-infiltrating lymphocytes (TILs) and the expression of MKL1 in breast cancer, we first evaluated the expressed fraction of TIL cells using the single sample Gene Set Enrichment Analysis (ssGSEA) algorithm by comparing the breast cancer gene expression matrix with those of the signatures from 22 reported TIL cell types [26]. Then, the relationship of the proportion matrix for the 22 TIL cells with MKL1 was calculated using Spearman’s correlation analysis. Another dataset in Gene Expression Omnibus (GEO) (GSE158309) [23], which contains 461 European breast cancer patients, was used for independent validation.

### Survival analysis of MKL1

We analyzed the correlation between MKL1 expression and survival status in breast cancer by grouping patients into high and low MKL1 expression groups. Progression-free survival and disease-free survival were used as endpoints. We used the Cox regression model in the survival and forest package to calculate and visualize the hazard ratio and Cox *p* values.

### Analysis of functional biopathway with MKL1

Spearman’s correlation analysis between MKL1 and other protein-coding genes was conducted. Top 300 MKL1 correlated genes were selected for further functional pathway analysis. We conducted pathway analyses by the Gene Ontology (GO) and Kyoto Encyclopedia of Genes and Genomes (KEGG) using the clusterPorfiler package. Pathways with false discovery rate <0.05 were retained.

### Prediction of chemosensitivity

The R package pRRophetic [27], based on the pharmacogenomics database of the Cancer Genome Project cell line data and the Cancer Cell Line Encyclopedia, was utilized to predict chemotherapeutic sensitivity in breast cancer patients by estimating the half-maximal inhibitory concentration (IC_50_). Default settings were used for the prediction model, including “whole blood” for reference tissue type selection and “cvFold = 10” for ridge regression model training.

### Statistical analysis

The Wilcoxon rank-sum test was used to detect differentially expressed MKL1 in different subgroups. We performed all the statistical analyses by R (v3.6.2). Two-sided *p* < 0.05 was considered statistically significant.

## RESULTS

### Distinct expression of MKL1 in different cancers

**FIGURE 1:**
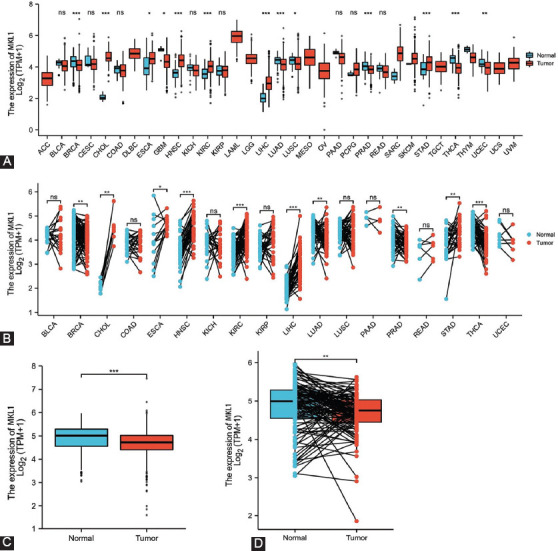
Megakaryocytic leukemia 1 (MKL1) expression levels of different human cancers in TCGA datasets. Non-paired (A) and paired (B) analyses revealed that MKL1 expression differed in various human cancers. Non-paired (C) and paired (D) analyses showed a significant difference of MKL1 expression levels between normal and breast cancer tissues. MKL1: Megakaryocytic leukemia 1, TGGA: The Cancer Genome Atlas.

The mRNA expression levels of MKL1 in different cancers were evaluated using TCGA dataset. Distinct expression of MKL1 in tumor and normal tissues is shown in Figure 1. According to both paired and non-paired analyses, MKL1 mRNA expression was lower in breast invasive carcinoma, lung adenocarcinoma, lung squamous cell carcinoma, thyroid carcinoma, and prostate adenocarcinoma. Higher MKL1 expression was indicated in cholangiocarcinoma, esophageal carcinoma, head-and-neck squamous cell carcinoma, kidney chromophobe, liver hepatocellular carcinoma, and stomach carcinoma (Figure 1A and B).

To investigate MKL1 expression in breast cancer, we examined both normal and breast tumor tissues using TCGA RNA-sequencing data. The non-paired analysis included 113 and 1109 normal and breast tumor tissues, respectively. A total of 112 paired normal and tumor tissues were evaluated using paired analysis. Both analyses showed that MKL1 expression was much lower in breast tumor tissues, indicating that MKL1 might be a candidate target in breast cancer patients (Figure 1C and D).

### Association between MKL1 expression and breast cancer patient survival

**FIGURE 2:**
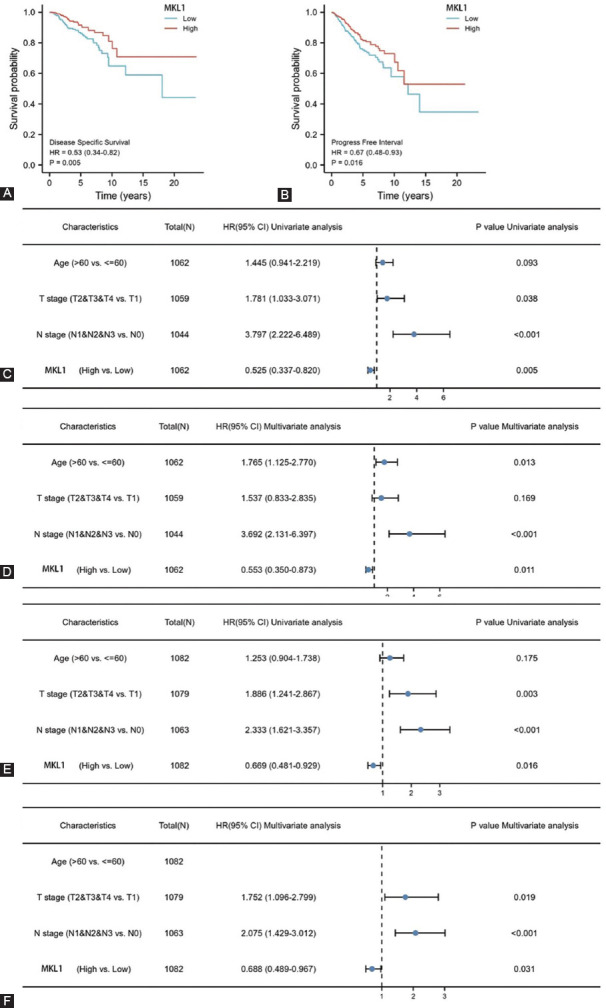
Survival analysis of breast cancer patients with different MKL1 expression levels. Kaplan–Meier analyses of DSS (A) and PFI (B) indicated that breast cancer patients with a higher MKL1 expression level would have a better survival. Both univariate (C) and multivariate (D) analyses of DSS revealed that the DSS of breast cancer patients was associated with N stage and MKL1 expression. Both univariate (E) and multivariate (F) analyses of PFI showed that the PFI of breast cancer patients was linked with T stage, N stage, and MKL1 expression. MKL1: Megakaryocytic leukemia 1, DSS: Disease-specific survival, PFI: Progression-free interval.

To explore the association between MKL1 expression and breast cancer patient survival, we analyzed the clinical characteristics of 1083 breast cancer patients (Table 1). A total of 541 and 542 patients were divided into two groups: Low and high MKL1 expression groups. Patients with high MKL1 expression were found to have a longer DSS (*p* = 0.005) and PFI (*p* = 0.016) (Figure 2A and B). Univariate and multivariate analyses of prognosis are shown in Figure 2C-F. This analysis revealed that T stage, N stage, and MKL1 expression were significantly associated with both DSS and PFI. These results demonstrate that MKL1 expression could predict the prognosis in patients with breast cancer.

**TABLE 1:**
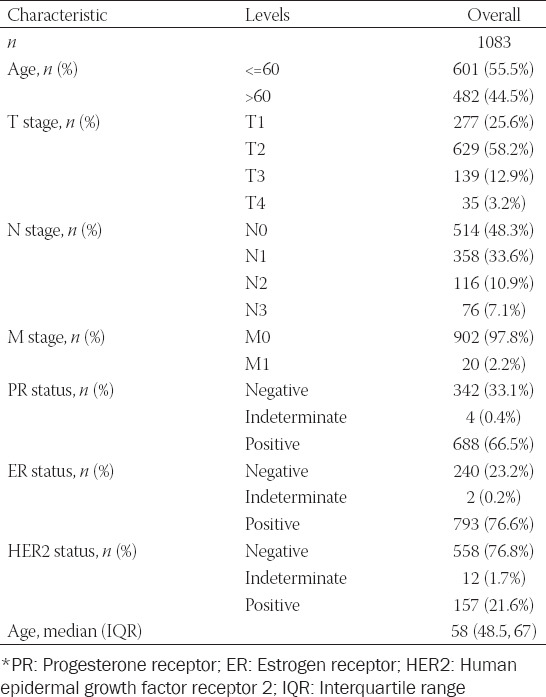
The clinical characteristics of 1083 breast cancer patients included in the analysis

### Association between MKL1 expression and the infiltration levels of immune cells

**FIGURE 3:**
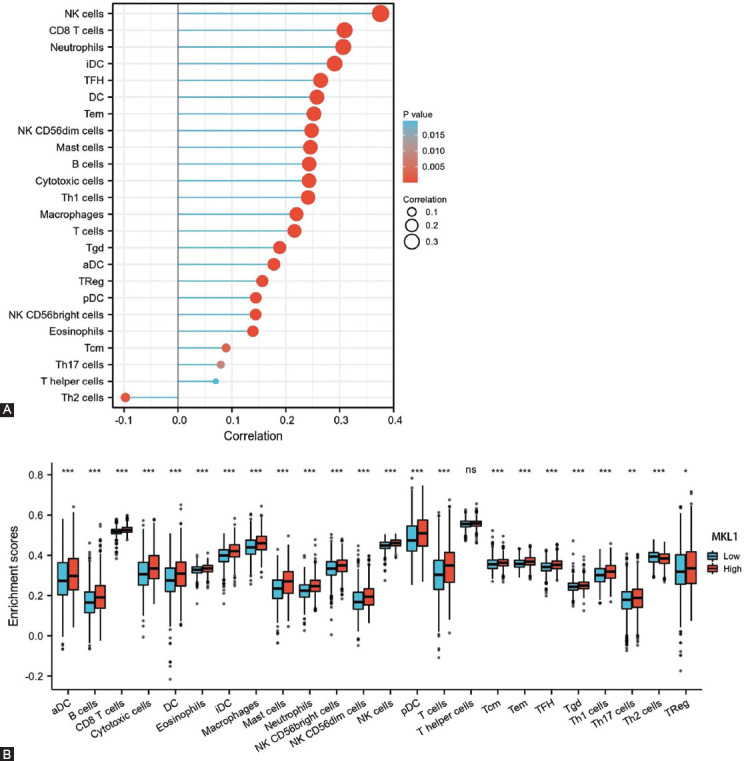
Correlation between MKL1 expression and the infiltration levels of immune cells. (A) Lollipop chart of the correlation between MKL1 and different infiltration levels of immune cells in breast cancer; (B) The enrichment of different tumor-infiltrating immune cells in breast cancer with low or high MKL1 expression. The MKL1 expression was positively linked with multiple infiltrating immune cells, including NK cells, CD8 T cells, neutrophils, DCs, and B cells. The negative correlation was only observed between MKL1 and Th2 cells. MKL1: Megakaryocytic leukemia 1, NK: Natural killer, DCs: Dendritic cells, T helper 2 cells.

Recently, tumor-infiltrating immune cells have been reported to influence the prognosis and survival in breast cancer patients. The relationship between MKL1 expression and the infiltration levels of immune cells in breast cancer was analyzed further analyzed [26,28]. MKL1 expression was positively correlated with almost all immune cells, except for T helper (Th) 2 cells (Figure 3A). Breast cancer with high MKL1 expression was found to have more infiltrating immune cells (Figure 3B), including activated DCs (*p* < 0.001), B cells (*p* < 0.001), CD8+ T cells (*p* < 0.001), cytotoxic cells (*p* < 0.001), DCs (*p* < 0.001), eosinophils (*p* < 0.001), immature DCs (*p* < 0.001), macrophages (*p* < 0.001), mast cells (*p* < 0.001), neutrophils (*p* < 0.001), natural killer (NK) CD56 bright cells (*p* < 0.001), NK CD56 dim cells (*p* < 0.001), NK cells (*p* < 0.001), plasmacytoid DCs (*p* < 0.001), T cells (*p* < 0.001), central memory T cells (*p* < 0.001), effective memory T cells (*p* < 0.001), follicular helper T cells (*p* < 0.001), gamma-delta T cells (*p* < 0.001), Th1 cells (*p* < 0.001), Th17 cells (*p* = 0.009), Th2 (*p* < 0.001), and regulatory T cells (Tregs) (*p* = 0.032). Our data suggest that high MKL1 expression would improve both innate and adaptive anti-tumor immune responses, which predict a good prognosis.

### Correlation analysis between MKL1 expression and seven types of infiltrating immune cells and immune marker sets

**FIGURE 4:**
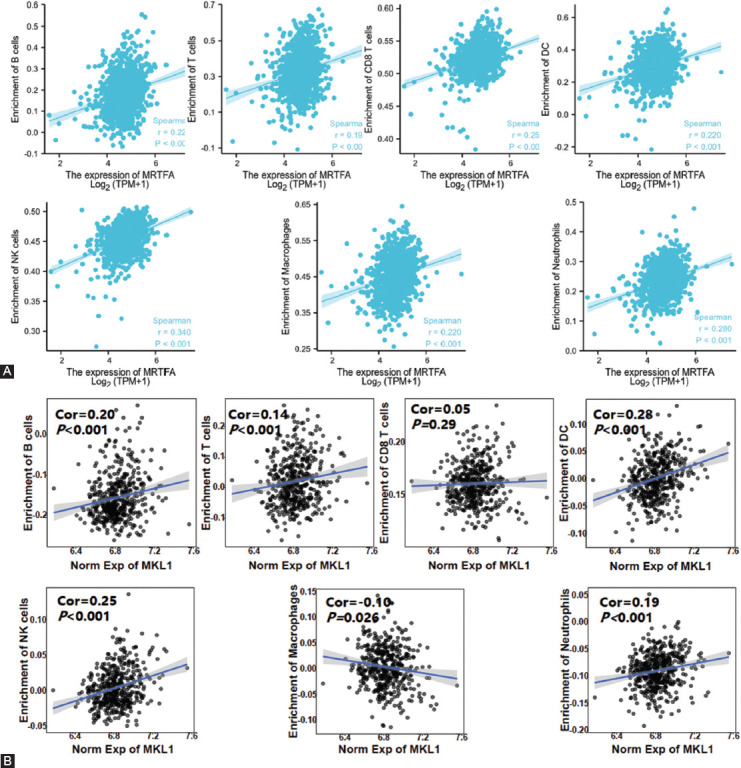
(A) The correlation between MKL1 expression and estimated fractions of B cells, T cells, CD8+ T cells, DCs, NK cells, macrophages, and neutrophils in TCGA breast cancer cohort. Positive correlations were observed between MKL1 expression and all these immune cells; (B) Validation of correlations between MKL1 and immune cell fractions in another breast cancer cohort GSE158309. MKL1: Megakaryocytic leukemia 1, NK: Natural killer, DCs: Dendritic cells, TGGA: The Cancer Genome Atlas.

Furthermore, we tested the correlation between MKL1 expression and seven types of infiltrating immune cells (B cells, T cells, CD8+ T cells, DCs, NK cells, macrophages, and neutrophils) in TCGA using ssGSEA. The results demonstrated that MKL1 expression was significantly correlated with B cells (r = 0.220, *p* < 0.001), T cells (r = 0.190, *p* < 0.001), CD8+ T cells (r = 0.250, *p* < 0.001), NK cells (r = 0.340, *p* < 0.001), DCs (r = 0.220, *p* < 0.001), macrophages (r = 0.220, *p* < 0.001), and neutrophils (r = 0.280, *p* < 0.001) (Figure 4A). To increase the credibility of our studies, another dataset in GEO (GSE158309) [23] was also employed to validate the correlation between MKL1 expression and these immune cells. Similar results were obtained as shown in Figures 4B.

**FIGURE 5:**
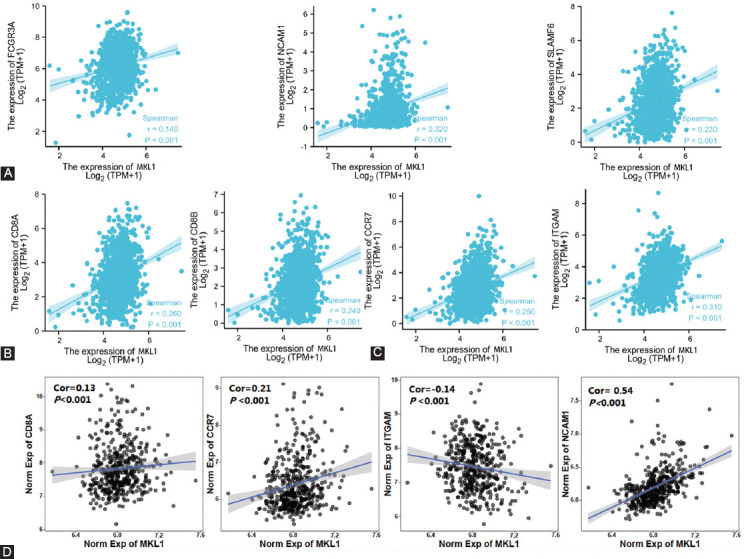
Correlation between MKL1 expression and reported immune cell surface markers. Positive correlations were found in (A) NK cells, (B) CD8+ T cells, and (C) neutrophils markers. (D) Validation of correlation in GSE158309. MKL1: Megakaryocytic leukemia 1, NK: Natural killer.

Considering that NK cells, CD8+ T cells, and neutrophils had a stronger correlation, we also examined the relationship between their specific cell surface markers and MKL1 expression. It was indicated that MKL1 expression also had a positive correlation with the markers of NK cells (Figure 5A), including Fc fragment of IgG receptor IIIa (r = 0.140, *p* < 0.001), neural cell adhesion molecule 1 (r = 0.320, *p* < 0.001), and SLAM family member 6 (r = 0.230, *p* < 0.001). CD8+ T cell markers, such as CD8A (r = 0.260, *p* < 0.001) and CD8B (r = 0.240, *p* < 0.001), were strongly associated with MKL1 expression (Figure 5B). C-C motif chemokine receptor 7 (r = 0.250, *p* < 0.001) and integrin subunit alpha M (r = 0.320, *p* < 0.001), the markers of neutrophils, also showed significant correlation with MKL1 expression (Figure 5C). Similar results were shown in analyses of GSE158309 (Figure 5D). Therefore, our findings suggest that MKL1 expression is associated with the infiltration levels of immune cells in breast cancer, revealing that MKL1 has a profound impact on regulating the compound and function of TME.

### Correlation analysis between MKL1 expression and immune control genes

**FIGURE 6:**
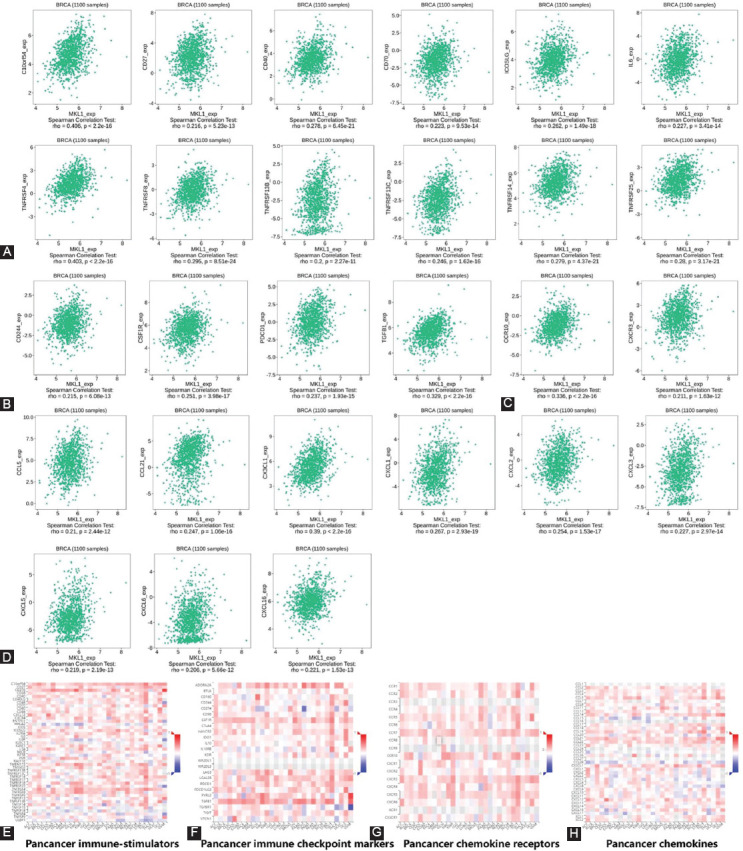
Correlation analysis of MKL1 expression with classic marker genes from (A) immune stimulators, (B) immune checkpoint markers, (C) chemokine receptors, and (D) chemokines in breast cancer samples. Pan-cancer level correlation analysis of MKL1 expression with marker genes from (E) immune stimulators, (F) immune checkpoint markers, (G) chemokine receptors, and (H) chemokines in breast cancer samples. MKL1: Megakaryocytic leukemia 1.

A novel therapy using immune checkpoint inhibitors (ICIs) has shown promising effectiveness in treating multiple cancers. To investigate the potential of MKL1 in immunotherapy, we analyzed the relationship between MKL1 and immune control genes. It has been shown that, in breast cancer, MKL1 was positively correlated with C100rf54, CD27, CD40, CD70, inducible T-cell costimulator ligand, interleukin (IL) 6, TNF receptor superfamily member (TNFRSF) 13 B, TNFRSF 13C, TNFRSF 14, TNFRSF 25, TNFRSF 4, and TNFRSF 8, all of which positively modulate the immune response (Figure 6A). In addition, MKL1 expression was associated with several immune checkpoint markers, including CD244, colony-stimulating factor 1 receptor, programmed cell death 1, and transforming growth factor beta 1 (TGFB1) (Figure 6B). These results indicate that MKL1 expression is linked with multiple immune control genes and may regulate the efficacy of ICIs in breast cancer.

### Correlation analysis between MKL1 expression and chemokines/chemokine receptors

To further explore the role of MKL1 in immune cell function and migration, we analyzed the correlation between its expression and chemokines and their receptors. MKL1 expression was positively correlated with C-C motif chemokine receptor 10 and C-X-C motif chemokine receptor 3 in breast cancer (Figure 6C). Chemokines, such as C-C motif chemokine ligand (CCL) 5, CCL 21, C-X3-C motif chemokine ligand 1, C-X-C motif chemokine ligand (CXCL) 1, CXCL 2, CXCL 3, CXCL 5, CXCL 6, and CXCL 16, were also upregulated when MKL1 expression level was increased (Figure 6D). These results imply that MKL1 expression is correlated with the expression of chemokines/chemokine receptors and could be a potential target for modulating immune cell function. Relationships between MKL1 expression, immune control genes, chemokines, and chemokine receptors in pan-cancer level were also explored as validation (Figure 6E-6H).

### Gene sets enriched in MKL1 expression phenotype

**FIGURE 7:**
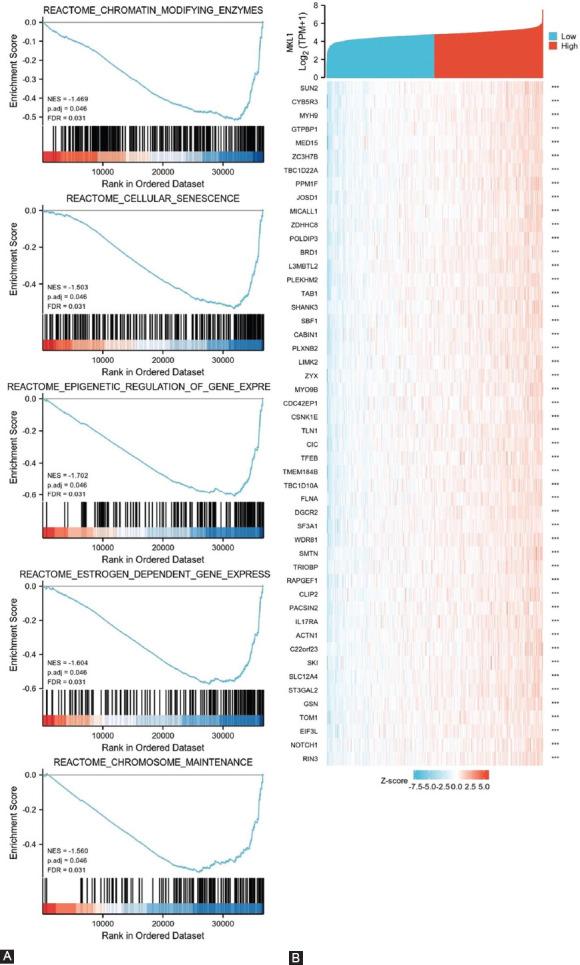
Gene set enrichment analysis of MKL1 in breast cancer. (A) Breast cancer with low MKL1 expression could activate five signaling pathways, such as chromatin-modifying enzymes, cellular senescence, epigenetic regulation of gene expression, estrogen-dependent gene expression, and chromosome maintenance; (B) Heatmap of related genes in breast cancer with different MKL1 expression. MKL1: Megakaryocytic leukemia 1.

To explore the function and related pathways of MKL1, a correlation analysis between MKL1 and other genes in breast cancer was performed using TCGA. The potential functional pathways based on the top 300 genes were analyzed by clusterProfiler R package. By analyzing GO and KEGG enrichment collection, GSEA was also employed to identify MKL1-related signaling pathways in these two groups. Five signaling pathways, including chromatin-modifying enzymes, cellular senescence, epigenetic regulation of gene expression, estrogen-dependent gene expression, and chromosome maintenance, were reported to be remarkably enriched in the low MKL1 expression group (Figure 7A). The top 50 genes that were most significantly connected with MKL1 are listed in a heatmap (Figure 7B). These results suggest the potential mechanism of MKL1 in modulating breast cancer development and the host immune response. Further studies are required to validate these findings.

### Drug sensitivity prediction

**FIGURE 8:**
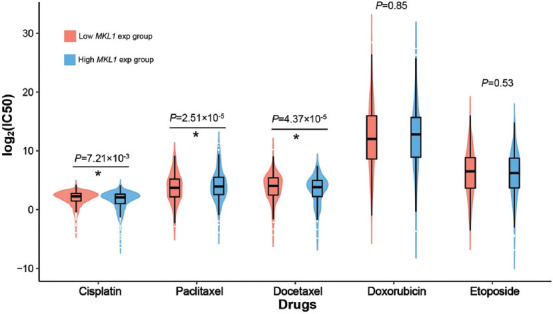
Drug sensitivity between high and low MKL1 expression group. The chemosensitivity to cisplatin, paclitaxel, and docetaxel was found different between high and low MKL1 expression groups. No differences were observed in doxorubicin and etoposide. IC_50_: Half maximal inhibitory concentration. MKL1: Megakaryocytic leukemia 1.

Drug chemosensitivity and/or drug resistance can influence the clinical outcomes of breast cancer treatment. Therefore, we explored the clinical implications in different MKL1 expression groups. We predict the individual drug sensitivities using a ridge regression model. As shown in Figure 8, two commonly used chemotherapy drugs in breast cancer therapy, cisplatin and docetaxel were associated with higher sensitivities in the low MKL1 expression group than in the high expression group (*p* = 7.21 × 10^−3^ for cisplatin and *p* = 4.37 × 10^−5^ for docetaxel). Conversely, paclitaxel was related to higher sensitivity in the high expression group (*p* = 2.51 × 10^−5^).

## DISCUSSION

Our study evaluated the MKL1 expression in breast cancer and its correlation with the behavior of the immune system and drug sensitivity. MKL1, which is critical in smooth muscle cell differentiation, interacts with the transcription factor myocardin and is indispensable for pathogenesis in a host of human diseases [29,30]. It helps transmit signals from the cytoskeleton to the nucleus and encodes nuclear protein at the same time. The fusion of MKL1 and the RNA-binding motif protein-15 gene is associated with a specific translocation event in acute megakaryocytic leukemia [31]. Recent studies have shown that MKL1 is important in the promotion of oncogenic development and progression, such as tumorigenesis, proliferation, autophagy, apoptosis, invasion, and metastasis. Furthermore, MKL1 has been linked to oncogenesis in non-small cell lung cancer, Hodgkin lymphoma, gastrointestinal tumor, hepatocellular carcinoma, and breast cancer, predominantly by specific transcription [20,22,32-35]. For example, TGF-β function is mediated by MKL1 in breast cancer, which induces RhoJ transcription to promote cancer cell invasion [35]. SAP-dependent MKL1 signaling is a novel mediator of malignant activity in mammary tumors related to radiotherapy and also provides a new method for therapy [36]. Our report showed that MKL1 expression was markedly higher in normal tissues than in breast invasive carcinoma. Increased MKL1 levels were linked with increased infiltration of immune cells and represented favorable prognosis in breast cancer patients.

With the development of ICIs, immunotherapy has become a promising therapeutic method for treating breast cancer [37]. The mortality rates in breast cancer have been prominently decreased by immunotherapeutic strategies, but there still remains a need to identify key genes, especially those that could modulate the compound of the TME [3,38]. Increasing evidence supports that immune cells within the TME could become a significant factor in predicting therapeutic efficacy and treatment outcomes [39-42]. In our study, we found that MKL1 was linked with the expression of different tumor-infiltrating immune cells. Indeed, MKL1 expression was positively correlated with the abundance of NK cells. NK cells can clear off cancer cells through cytolytic granules, cytokines, and chemokines. A recent study showed that conventional type 1 DCs recruited by tumor-resident NK cells could promote anti-tumor immunity. Tumor cells release prostaglandin E2, which destroys the function of NK cells and leads to immune escape at the same time [43,44]. NK cell activation can detect tumor cells, leading to tumor cell apoptosis [45]. The aggressive biological behavior of triple-negative breast cancer is inhibited through IL-6 secreted by infiltrating NK cells [46]. It has also been reported that low NK cell infiltration could predict poor survival [47]. In addition, decreased NK cell infiltration may be a predictive marker of breast cancer chemotherapy failure [48].

CD8+ T cells are also critical components of the tumor immune microenvironment. Our study indicated that breast cancer patients with higher MKL1 expression had more tumor-infiltrating CD8+ T cells. Baker et al. demonstrated that CD8+ T cells were significant in predicting prognosis in estrogen receptor (ER)-negative breast cancer [49]. A low CD8+ T cell score is associated with poor survival in triple-negative breast cancer [50]. Moreover, it has also been reported that increasing MKL1 levels make cancer cells more vulnerable to cytotoxic T cells and NK cells [51]. Therefore, MKL1 might modulate the functions of CD8+ T cells and has the potential to become a biomarker for immunotherapy in breast cancer.

According to our results, MKL1 expression was positively associated with the infiltration levels of T cells (particularly CD8+ T cells), neutrophils, DCs, B cells, macrophages, and NK cells. MKL1 expression was also linked to markers of several immune genes. All the results implicated the remarkable role of MKL1 in regulating the tumor immune microenvironment in breast cancer.

MKL1 is a cofactor that regulates epigenetic transcription. In our study, GSEA showed that chromatin-modifying enzymes, cellular senescence, epigenetic regulation of gene expression, estrogen-dependent gene expression, and chromosome maintenance were differentially enriched in MKL1 low expression phenotype. Further research revealed that H3K4 methyltransferase is recruited by MKL1, which potentiates cell migration and invasion through matrix metallopeptidase 9 in cancer [18]. Epigenetic mechanisms are considered to regulate numerous aspects of breast cancer, including tumor growth, invasion, and immune response to the microenvironment. Furthermore, cancer cells are exposed to attacks by the immune system under epigenetic modulation, which increases their sensitivity to immunotherapy [52-56]. Recently, several epidrugs, such as histone deacetylases, have been approved for ER+ breast cancer development, recurrence, and drug resistance [57,58]. Epigenetic regulation is a potential strategy to combat breast cancer in the future.

Drug sensitivity analysis showed appropriate chemotherapeutic drugs for different MKL1 expression groups. The group with high expression was sensitive to paclitaxel, while the low abundance group showed potential sensitivity for cisplatin and docetaxel. Multiple chemotherapeutics have been reported to enhance anticancer immune responses and regulate the functions of several immune cells [59]. For example, paclitaxel can promote the maturation and activation of DCs through Toll-like receptor 4 and stimulate the secretion of IL-12, which can activate T-cell function [60]. A clinical trial evaluated the efficacy of paclitaxel in the neoadjuvant treatment of breast cancer patients, revealed that paclitaxel could increase tumor-infiltrating lymphocytes and provide better survival [61]. Similar to paclitaxel, docetaxel was also found to decrease the number of all subtypes of Tregs both *in vitro* and *in vivo* [62]. Considering that MKL1 expression is correlated with infiltrating NK cells, DCs and Tregs, this may explain the difference in paclitaxel and docetaxel sensitivity in patients with different MKL1 expression. These results showed the probability of MKL1 as a chemotherapeutic indicator in clinical treatments, and further studies are still needed to validate its specific roles.

However, we acknowledge the underlying limitations of our study. The whole study was performed using public databases, and more experimental verification is necessary both *in vivo* and *in vitro*.

## CONCLUSION

Our study suggests that MKL1 could serve as a valuable prognostic biomarker associated with immune cell infiltration and chemotherapeutic drug sensitivity in breast cancer. We need to validate the role of MKL1 in breast cancer further.
